# Determining the Impact of a Community-Based Intervention on Knowledge Gained and Attitudes Towards the HPV Vaccine in Virginia

**DOI:** 10.1007/s13187-022-02169-5

**Published:** 2022-04-23

**Authors:** Reanna Panagides, Noelle Voges, Janaye Oliver, Debbie Bridwell, Emma Mitchell

**Affiliations:** 1 University of Virginia, School of Nursing, Charlottesville, VA USA; 2 University of Virginia, Cancer Center, Charlottesville, VA USA; 3 Virginia Department of Health, Richmond, VA USA

**Keywords:** HPV vaccination, Human papillomavirus, Community-based interventionl, Rural

## Abstract

HPV vaccination series completion rates have increased steadily in the USA, yet specific areas continue to be burdened by HPV-related cancers, including rural areas of Virginia. The primary purpose of this study is to compare the impact of an community driven educational film about HPV on intention to vaccinate and knowledge gained in urban and rural areas of Virginia. From October 2016 to September 2019, the CDC-approved documentary “Someone You Love: The HPV Epidemic” was screened and followed by a Q&A session. Intention to vaccinate and knowledge gained after seeing the DVD intervention were measured through a pre-post-survey and analyzed data using chi-squared tests. The sample included males and females of all races and ages 18+ that identified as either student, parent/guardian, and/or healthcare provider from rural and urban geographical areas. Changes in knowledge about HPV were statistically significant in two out of seven questions (*p* < 0.05). Changes in attitude were statistically significant in every attitude-based question about HPV (*p* < 0.05). There were significant differences in knowledge gained and attitudes towards the HPV vaccine when comparing urban and rural locations as well. More research is needed to explore the efficacy of community-based interventions to increase uptake and series completion of HPV vaccination, particularly in rural areas most impacted by HPV-associated cancers.

**Supplementary Information:**

The online version contains supplementary material available at 10.1007/s13187-022-02169-5.

## Introduction

In the USA, 14 million people are infected with Human Papillomavirus annually, making it the most common sexually transmitted infection [1]. In addition to cervical cancer, HPV is known to cause cancer of the throat, vulva, penis, and anus [1]. Each year, about half of all new HPV infections occur in adolescents and young adults 15–24 years old [1]. The HPV vaccine inhibits HPV from spreading and is an effective method in preventing cervical, anal, penile, and oropharyngeal cancers [2]. Although safe and effective, it is often underutilized due to the associated stigma and misinformation spread about the vaccine [2]. In 2017, USA’s vaccination rates for adolescents (13–17) were 65.5% for the first dose and 48.6% for the entire series [1]. In 2017, Virginian vaccination rates of female adolescents completing the series were 49.5% and 37.5% for male adolescents [3].

Rural communities, which are defined as areas that contain less than 2,500 people, of which less than 1,500 reside outside institutional group quarters [4], such as Appalachia and Southwest Virginia, have higher rates of HPV-related diseases [3]. Adolescents in rural areas are less likely to be vaccinated against HPV cancers than teens in urban areas [1]. The 2016 Center for Disease Control National Immunization Survey-Teen (NIS-Teen) reported 70.1% of teens living in the metropolitan statistical area (MSA) principal cities had received one or more doses of the HPV vaccine, compared to 59.3% of teens living in non-MSA areas [1]. Unlike urban areas, there exists limited access to providers and health care services in rural areas which contributes to these disparities in vaccination initiation rates. In a study of un-vaccinated adolescents in rural counties, one of the most common barriers, in addition to lack of information about the vaccine, was the lack of a strong recommendation from their primary care provider [3].

Provider recommendation is a key part of vaccination initiation because it serves as a cue to action for many patients to participate in a healthy behavior. Although this is an important predictor of HPV vaccine series completion, 66% of physicians report that they do not have enough time to educate parents and adolescents about the vaccine [3]. A community-based approach may be effective in combating some of these barriers because they do not rely on patients attending clinic appointments. Community-based interventions like an education film, radio ad, and social media campaign serve as a cue to action for patients that have not been recommended to receive a vaccine from the primary care provider. Such interventions specifically target barriers that involve lack of education/misinformation and lack of time for education on behalf of the provider because they can be organized and implemented by nurses, teachers, counselors, or public health workers that reside within or outside these communities. Community-based education interventions have no restraint on time or location and can therefore be more effective in communicating the importance of the vaccine and addressing misinformation. Therefore, community-based education interventions could be more effective in rural areas when compared to urban areas.

To date, much of the HPV vaccine-related research has focused on individual interventions, interventions with parents, and interventions with providers, but there is a lack of community-based intervention research. In addition, there are very few studies that compare community-based interventions in urban and rural areas. Although understudied, community-based interventions may be more beneficial in rural areas than urban areas due to limited access to education, fewer providers, and stigma as mentioned previously. It is important to determine whether community-based education interventions are more or less effective in rural areas than urban areas for the sake of being cost-effective and efficient in our effort to influence public health.

Although limited, there are a few community-based education intervention research studies that aim to increase HPV vaccine uptake or intention to uptake in rural or urban areas in the past 10 years. Community-based interventions include videos, vaccine fairs, radio or newspaper ads, websites, social media campaigns, school-based reminders, educational sessions, and radionovelas [5–14]. Of the 10 interventions represented in the literature, 8 were conducted in rural settings and 9 had significant results in efficacy which was measured by vaccine uptake, intention to vaccinate, knowledge gained, and/or perception of reduced barriers to vaccinating. There were no studies that compared the efficacy of a community-based intervention in rural versus urban locations.

### Purpose

The literature has shown that utilization of community-based education interventions improves willingness to vaccinate and knowledge gained regarding HPV-related information [15]. Studies have also shown that education interventions decreased several perceived barriers to receiving the HPV vaccine [16]. The primary purpose of this study is to compare the impact of an educational film intervention (as a cue to action) on HPV intention to vaccinate and knowledge gained in urban and rural areas. The secondary purpose of this study is to ultimately increase knowledge and intent to receive the HPV vaccine.

## Methods

Through an initiative led by the Indiana Immunization Coalition, the Virginia Department of Health (VDH) conducted multiple screenings of the “Someone You Love: The HPV Epidemic” film in urban and rural areas of Virginia. This film was used as the community-based intervention for this study. The Someone You Love (SYL) documentary follows the lives of 5 women diagnosed with cervical cancer caused by the HPV virus, highlighting the women’s diagnosis and addressing misinformation about and the stigma surrounding HPV [17]. An electronic or paper pre/post-survey was distributed to determine changes in knowledge, attitudes, and beliefs regarding the HPV vaccine. The survey was adapted from the SYL Toolkit produced by the Ohio Partners for Cancer Control. Comparative statistics between urban and rural locations where the film was shown were used to determine the efficacy of this community-based intervention within different populations. IRB approval was obtained before data collection and participants were not compensated for their time (UVA SBS-IRB#3158).

### Design

**Fig. 1:**
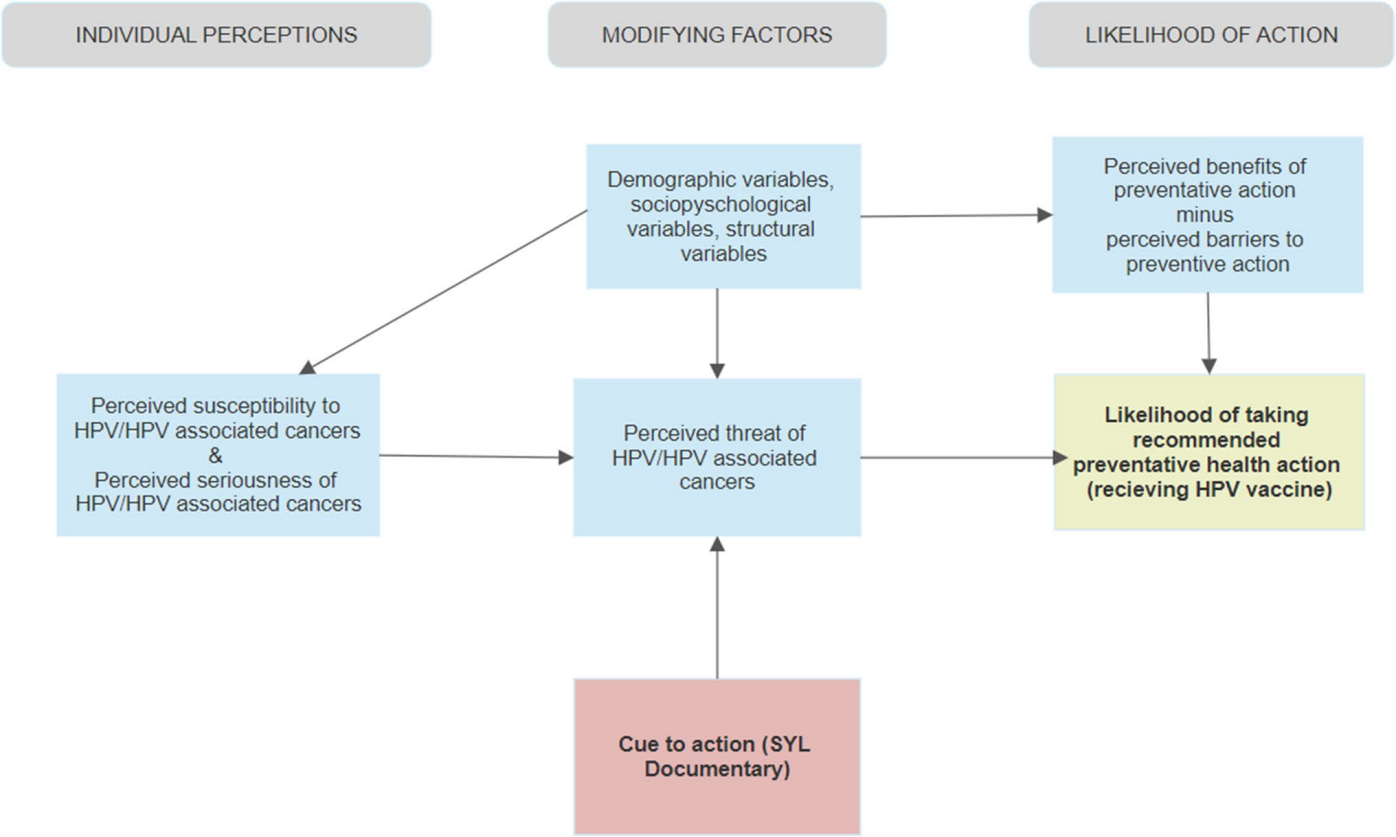
Health belief model adapted to include SYL intervention [19]

According to the Health Belief Models (HBM) theoretical model, disease prevention techniques are used when individuals have an increased perceived susceptibility, an increased perceived severity, are motivated, usually by a cue to action, to change their behavior, and have decreased perceived barriers [18]. The Someone You Love documentary acts as a cue to action to change behavior that includes both information about HPV, HPV transmission, the risk of contracting HPV/serious complication caused by the virus, and HPV prevention methods (i.e., the HPV vaccine) (Fig. 1).

Based on the Health Belief Model (HBM), outcomes of this cue to action include changes in knowledge, attitudes, and beliefs regarding the HPV vaccine. Pre-post-surveys included demographics, knowledge, attitudes, and beliefs regarding HPV vaccination to measure these anticipated outcomes.

### Sample and Setting

The study population included participant’s ages 18 + , male and female, and all races. Recruitment was solely from those who chose to attend the SYL viewing event. The event was promoted through flyers and email communication. Emails were sent to social, academic, and civic groups to share with their members. The study was conducted in academic institutions throughout Virginia and at the Virginia Department of Health. There were a total of seven geographic areas studied (4 rural and 3 urban) [4]. Rural and urban areas were defined through a website that uses data from the US Census Bureau, the Office and Management and Budget, and the Economic Research Service o the US Department of Agriculture [4]. To quantify as an urban area, the area must contain at least 2,500 people, of which at least 1,500 of which reside outside institutional group quarters [4]. To quantify as rural, it merely contains all populations, housing, and territory not included within this previously defined urban area [4].

### Procedures and Intervention

Data collection began in October 2016 and ended in the Spring of 2019. This research study consisted of a pre- and post-survey. Before showing the documentary, the study team explained the purpose of the pre- and post-survey and then asked for opt-in consent. The opt-in consent procedure consisted of the researcher reading aloud the consent form to the participants. Participants were asked to use their smartphones to access the provided link projected on the screen which took them to the Qualtrics highly sensitive data portal to begin the pre-survey. After viewing the documentary, the post-survey was administered through the same process. Participants who took the survey through Qualtrics were assigned a numeric code randomly generated through the Qualtrics software which was used to link the pre- and the post-survey. Participants who took the survey on paper did not receive a linking numeric code although all other procedures were conducted the same. This is an important detail to note with regard to how the data was analyzed.

### Measures

The effectiveness of the film intervention was measured by analyzing the differences in the mean responses between the pre/post-survey results in knowledge gained, attitudes towards, and intention towards receiving the HPV vaccine. Although actual uptake of the HPV vaccine would be the best indicator of the effectiveness of the film intervention, intention to vaccinate is significantly associated with uptake [20].

### Data Analysis Strategy

Data analysis was performed using the Statistical Package for the Social Sciences (SPSS). Descriptive statistics were used to analyze the demographics of participants. Chi-squared tests were used to determine the difference in the means between the pre- survey and the post-survey for knowledge-based questions and attitude-based questions in a majority of the data set. A sub-analysis was performed for a small part of the data set that had linking identifiers between the pre- and the post-surveys. Sub-analyses were conducted to compare changes in knowledge and attitudes in part of the data set that had linking IDs, to compare responses between the rural and urban intervention locations, and to compare means between the pre- and post-survey in the data set that excluded participants that identified as healthcare professionals. Due to the nature of the statistical tests used to analyze this data, these sub-analyses were only conducted on the linked data (Qualtrics surveys) set rather than the entire data set (paper surveys).

## Results

### Main Data Set Analysis

With regard to the main data set, there was a total of 149 who completed the pre-survey with a median of 48 years of age. The majority of participants was female (84.1%, *n* = 127), from a rural geographical area (57%, *n* = 86), healthcare providers (56.3%, *n* = 85), and Caucasian (84.1%, *n* = 127) (please see table 1 for a further breakdown of our sample population). Correct responses regarding knowledge about HPV between the pre- and the post-survey were significantly different in four out of seven questions in the sample. The following question is as listed: (1) HPV is a rare, sexually transmitted disease, (2) You can only get HPV through vaginal sex, (3) How to prevent transmission of HPV (It is important to note that this was the only question that the post-survey mean was significantly lower than the pre-survey mean), and (4) What types of cancers are associated with HPV (please refer to Table 2 for a further breakdown of the results).

There were statistically significant changes in every attitude question. Attitude questions included (1) How safe do you think the vaccine is that prevents HPV? (2) How important do you think it is for people between the ages of 9 and 26 to be vaccinated to prevent HPV? (3) If you are a healthcare provider, please answer the following question: How likely are you to talk to your patients about HPV? (4) If you are NOT a healthcare provider, please answer the following question: How likely are you to talk to your/your child’s doctor about HPV? Please refer to Table 3 for a further breakdown of the results.

### Linked Data Set Sub-analysis

With regard to the linked data set, there were a total of 38 participants which had matching pre- and post-surveys with a linking ID. The majority of participants were female (86.8%, *n* = 33), students (68.4%, *n* = 26), and Caucasian (89.5%, *n* = 37). Regarding vaccine initiation/completion rates, 34% (*n* = 30) participants had received three shots in the HPV vaccine series (please refer to Table 4 for a further breakdown of the results).

For the linked data, one knowledge-based question produced significant results, “what cancers are associated with HPV?” For the linked sub-analysis, a Wilcoxon signed-rank test was used to analyze the Likert style questions for this sub-analysis because the sample was skewed and the pre- and the post-tests were linked. Using the Wilcoxon signed ranks test, two out of six questions produced significant results: (1) Do you plan to get the vaccine in the next 6 months?” and (2) “How important do you think it is for people between the ages of 9 and 26 to be vaccinated to prevent HPV?” (please refer to tables 5 and 6 for a further breakdown of the results).

### Students and Parents/Guardians (Excluding Healthcare Professionals) Sub-analysis

A sub-analysis that excluded healthcare professionals from the linked data set was conducted to explore the knowledge and attitude change of just students and parents/guardians. This analysis recognizes that the healthcare providers pre-existing knowledge about HPV might skew the results and therefore were eliminated for this sub-analysis. There were statistically significant changes in one knowledge-based question and two attitude-based questions. The knowledge-based question was in regard to the types of cancers associated with HPV. The attitude-based questions included (1) how safe do you think the vaccine is that prevents HPV? and (2) How likely are you to talk to you/your child’s doctor about HPV? (please refer to Tables 7 and 8 for a further breakdown of the results).

### Rural Versus Urban Sub-analysis

There were significant changes in two knowledge-based questions and three attitude-based questions when analyzing the results of the urban sites only. Knowledge-based questions include (1) HPV is a rare, sexually transmitted disease, (2) What are some ways to prevent the spread of HPV? (3) How safe do you think the vaccine is that prevents HPV? Attitude-based questions include (1) How safe do you think the vaccine is that prevents HPV? (2) How important do you think it is for people between the ages of 9 and 26 to be vaccinated to prevent HPV? and (3) How likely are you to talk to your patients about the HPV vaccine? (please refer to Table 9 for a further breakdown of the results.

There were significant changes in one knowledge-based question and two attitude-based questions when analyzing the results of the rural sites only. The knowledge-based question was in regard to types of cancer associated with HPV. The attitude-based questions included (1) how safe do you think the vaccine is that prevents HPV and (2) how likely you are to talk to your/your child’s doctor about the HPV vaccine? (please refer to table 10 for a further breakdown of the results).

## Discussion

Similar to the studies about HPV-related education interventions in community-based settings in the literature [5–14], this film-based education intervention produced significant results in increasing knowledge gained and changing attitudes toward the HPV vaccine. Accessing HPV information through a film intervention specifically targets barriers that involve lack of education/misinformation and lack of time for education on behalf of the provider [3]. This type of intervention could be utilized in rural Virginia not only because this population is at greater risk for HPV-related cancers, but because the film intervention can be conducted anywhere in the community. This will be valuable in locations that have limited access to primary care providers and clinics. This is also especially important in the context of the pandemic, as it continues to allow information to be accessible to communities during a time of social isolation in rural and urban areas alike. Since the COVID pandemic, this intervention has been adapted to a virtual format which includes a live Q&A after the film.

Unlike previous studies, we were able to compare survey responses between the urban and rural locations where the intervention was implemented. There were limitations to the study. First, the majority of the data collected was unlinked; therefore, researchers compared the means of the pre- and the post-surveys rather than analyzing the differences in the pre/post-pairs. Linking the pre- and the post-surveys for future data collection will allow us to use more individualized analyses and allow us to compare more subgroups in our data set. Another limitation of the study is that the film solely focused on female narratives. Including a male narrative which focuses on either oral, anal, or penile cancer would be one way to increase knowledge regarding the perception of risk in regard to HPV-associated cancers. In addition, there was very limited racial diversity within the study sample. It is important to note that the findings of this study are not generalizable to the racially marginalized communities.

## Conclusion

This study measured the impact of an educational film intervention on HPV intention to vaccinate and knowledge gained in urban and rural areas of Virginia. There were statistically significant changes in knowledge gained and HPV intention to vaccinate in the pre- and the post-survey. After the film intervention, participants indicated that they felt more at risk for HPV infection and subsequent cancers and that they felt more comfortable either talking to their provider/child’s provider about the vaccine (if they did not identify themselves as a healthcare provider). Participants also indicated that they felt more comfortable talking to their patients about the HPV vaccine if they identified as a healthcare provider. Although previous studies show that community-based interventions have been successful in increasing knowledge about the HPV vaccine and uptake of the vaccine [5–14], this study analyzed the impact of a video-intervention and compared these findings in rural and urban study locations. Rural locations had a significant increase in the question pertaining to knowledge about cancers specifically associated with HPV.

This analysis supports future efforts utilizing videos as community-based interventions to promote HPV vaccination within rural areas. This style of video intervention may be specifically successful if distributed to rural physicians or parent/guardian within schools as a way to increase HPV vaccination education and uptake. This type of video may also be easily incorporated into school-based sexual and reproduction health education curriculum as well. Due to the ease of video creation and distribution, future researchers or healthcare professionals should consider developing an intervention that targets specific populations and addresses cultural barriers in that region to obtaining the HPV vaccine. More research is needed to explore the efficacy of community-based interventions to increase actual uptake and series completion of the HPV vaccine, particularly in rural areas, male, and racial diverse populations. In the context of COVID-19, when HPV vaccination rates have significantly decreased [21, 22], innovative community-based interventions to promote HPV vaccination, like this one, remain critical. Researchers must continue to explore virtual and other platforms to reach this vulnerable, underserved, and undervaccinated audience.

## Supplementary Information

Below is the link to the electronic supplementary material.

Supplementary file1 (DOCX 58 KB)

## Data Availability

Not applicable.
