# Supplementary material for: Determining the Impact of a Community-Based Intervention on Knowledge Gained and Attitudes Towards the HPV Vaccine in Virginia
Source: J Cancer Educ. 2022 Apr 23;38(2):646–51. doi: 10.1007/s13187-022-02169-5 (PMC9034253; doi:10.1007/s13187-022-02169-5)
Supplement: Supplementary file 1 — Supplementary file1 (DOCX 58 KB) [file 13187_2022_2169_MOESM1_ESM.docx]

**Appendix**

**MAIN DATA SET**

Table 1. Demographic/Descriptive Characteristics

Pre-Survey: n=200

| **Characteristic** | ***n*** | **%** |
| --- | --- | --- |
| Gender | 28 | 14 |
| Female | 170 | 85 |
| Male |  |  |
| Location |  |  |
| Urban | 65 | 32.5 |
| Rural | 135 | 67.5 |
| Profession |  |  |
| Student | 44 | 22 |
| Parent/Guardian | 45 | 22.5 |
| Healthcare Providers | 104 | 52 |
| Other | 29 | 14.5 |
| Race/Ethnicity |  |  |
| White | 170 | 85 |
| Black | 14 | 7 |
| Asian | 7 | 3.5 |
| American Indian or Alaskan native | 0 | 0 |
| Hispanic | 8 | 4 |

Post-Survey: n=171

| **Characteristic** | ***n*** | **%** |
| --- | --- | --- |
| Gender |  |  |
| Female | 148 | 86.5 |
| Male | 17 | 9.9 |
| Location |  |  |
| Urban | 55 | 32.2 |
| Rural | 116 | 67.8 |
| Profession |  |  |
| Student | 35 | 20.5 |
| Parent/Guardian | 45 | 26.3 |
| Healthcare Providers | 91 | 53.2 |
| Other | 25 | 14.6 |
| Race/Ethnicity |  |  |
| White | 150 | 87.7 |
| Black | 8 | 4.7 |
| Asian | 1 | 0.6 |
| American Indian or Alaskan native | 3 | 1.8 |
| Hispanic | 9 | 5.3 |

Table 2. Significant Changes in Knowledge by Question

| **Knowledge Questions** | **% correct in Presurvey** | **% correct in Postsurvey** | **χ²** | **p** |
| --- | --- | --- | --- | --- |
| HPV is a rare, sexually transmitted disease. | 82.9 | 92.4 | 7.471 | <0.05 |
| What types of cancers are associated with HPV | 78.5 | 95.9 | 23.952 | <0.05 |

Table 3. Significant Changes in Attitudes by Question

| **Attitude Questions** | **χ²** | **p** |
| --- | --- | --- |
| How safe do you think the vaccine is that prevents HPV? | 24.668 | <0.05 |
| How important do you think it is for people between the ages 9 and 26 to be vaccinated to prevent HPV? | 11.537 | <0.05 |
| If you are a healthcare provider, please answer the following question: How likely are you to talk to your patients about HPV? | 17.777 | <0.05 |
| If you are NOT a healthcare provider, please answer the following question: How likely are you to talk to your/your child’s doctor about HPV? | 21.848 | <0.05 |

**LINKED DATA SUB-ANALYSIS**

Table 4. Demographic/Descriptive Characteristics in Sub-Analysis

| **Characteristic/behavior** | ***n*** | **%** |
| --- | --- | --- |
| Gender |  |  |
| Female | 33 | 86.8% |
| Male | 5 | 13.2% |
| Profession |  |  |
| Student | 26 | 68.4% |
| Parent/Guardian | 4 | 10.5% |
| Healthcare Provider | 16 | 42.1% |
| Other | 3 | 7.9% |
| Race/Ethnicity |  |  |
| White | 37 | 89.5% |
| Black | 1 | 2.6% |
| Asian | 5 | 10.2% |
| American Indian or Alaskan native | 3 | 7.9% |
| Hispanic | 2 | 5.3% |
| Received the HPV vaccine | 42 | 48.3% |
| HPV vaccine series # of shots |  |  |
| 1 | 2 | 2.3% |
| 2 | 4 | 4.6% |
| 3 | 30 | 34.5% |
| Unsure how many shots | 6 | 6.9% |

Table 5. Significant Changes in Knowledge by Question in Sub-Analysis

| **Knowledge Questions** | **% correct in Presurvey** | **% correct in Postsurvey** | **χ²** | **p** |
| --- | --- | --- | --- | --- |
| What cancers are associated with HPV | n/a | n/a | 5.859 | <0.05 |

Table 6. Changes in Attitudes by Question in Sub-Analysis

| **Attitude Questions** | **Z** | **p** |
| --- | --- | --- |
| Do you plan to get the vaccine in the next 6 months? | -2.318 | <0.05 |
| How important do you think it is for people between the ages of 9 and 26 to be vaccinated to prevent HPV? | -2.320 | <0.05 |

**STUDENTS and PARENTS/GUARDIANS (HEALTHCARE PROFESSIONALS EXCLUDED) SUB-ANALYSIS**

Table 7. Significant Changes in Knowledge in Sub-Analysis

| **Knowledge Questions** | **% correct in Presurvey** | **% correct in Postsurvey** | **χ²** | **p** |
| --- | --- | --- | --- | --- |
| What types of cancers are associated with HPV | 65.6 | 95.0 | 22.678 | <0.05 |

Table 8. Changes in Attitudes by Question in Sub-Analysis

| **Attitude Questions** | **χ²** | **p** |
| --- | --- | --- |
| How safe do you think the vaccine is that prevents HPV? | 19.619 | <0.05 |
| If you are NOT a healthcare provider, please answer the following question: How likely are you to talk to you/your child’s doctor about HPV? | 20.358 | <0.05 |

**RURAL v. URBAN SUB-ANALYSIS**

Table 9. Significant Changes in Responses in Urban Sub-Analysis

| **Question** | **%correct pre survey** | **%correct post survey** | **χ²** | **p** |
| --- | --- | --- | --- | --- |
| HPV is a rare, sexually transmitted disease. | 89.1 | 100.0 | 6.392 | <0.05 |
| What are some ways to prevent the spread of HPV? | 81.5 | 65.5 | 4.022 | <0.05 |
| How safe do you think the vaccine is that prevents HPV? | n/a (attitude question) | n/a (attitude question) | 8.618 | <0.05 |
| How important do you think it is for people between the ages of 9 and 26 to be vaccinated to prevent HPV? | n/a (attitude question) | n/a (attitude question) | 7.562 | <0.05 |
| If you are a healthcare provider, please answer the following question: How likely are you to talk to your patients about the HPV vaccine? | n/a (attitude question) | n/a (attitude question) | 12.833 | <0.05 |

Table 10. Significant Changes in Responses in Rural Sub-Analysis

| **Question** | **%correct pre survey** | **%correct post survey** | **χ²** | **p** |
| --- | --- | --- | --- | --- |
| What types of cancers are associated with HPV | 74.8 | 95.7 | 20.716 | <0.05 |
| How safe do you think the vaccine is that prevents HPV? | n/a (attitude question) | n/a (attitude question) | 17.409 | <0.05 |
| If you are NOT a healthcare provider, please answer the following question: How likely are you to talk to your/your child’s doctor about HPV? | n/a (attitude question) | n/a (attitude question) | 15.949 | <0.05 |
